# The Development of New SSR Markers and an Assay for Genotyping Sweet Cherry (*Prunus avium* L.) in One Reaction

**DOI:** 10.3390/ijms27052324

**Published:** 2026-03-01

**Authors:** Jana Čmejlová, Kateřina Holušová, Boris Krška, Pavol Suran, Jan Bartoš, Radek Čmejla

**Affiliations:** 1 Research and Breeding Institute of Pomology Holovousy Ltd., Holovousy 129, 508 01 Holovousy, Czech Republic; jana.cmejlova@vsuo.cz (J.Č.); boris.krska@vsuo.cz (B.K.);; 2 Institute of Experimental Botany, Czech Academy of Sciences, Centre of Plant Structural and Functional Genomics, Šlechtitelů 31, 779 00 Olomouc, Czech Republic; holusovak@ueb.cas.cz (K.H.); bartos@ueb.cas.cz (J.B.)

**Keywords:** multiplex fragment analysis, population analysis, parentage analysis

## Abstract

Sweet cherry (*Prunus avium* L.) exhibits relatively low genetic diversity because of the self-compatibility of some varieties and repeated crossings of the same genotypes. High-quality markers are therefore needed for their reliable discrimination. However, the most currently used simple sequence repeat (SSR) markers offer only limited resolution for genotyping purposes. Here, thirty new highly polymorphic SSR markers were extracted from whole-genome sequences of 299 sweet cherry genotypes. Then, 16 highly polymorphic SSR markers were selected, multiplexed into one PCR, and successfully verified on a collection containing 294 unique genotypes. Compared with the set of 16 SSR markers recommended by the European Cooperative Programme for Plant Genetic Resources (ECPGR) for sweet cherry genotyping, our newly developed system has a seven orders of magnitude lower probability of the random identity of two genetically distinct samples than the ECPGR set (10^−19^ vs. 10^−12^). This higher resolution not only enables more precise genotyping but can also be successfully used for parentage or population analyses. This new and unique one-tube approach for sweet cherry genotyping will substantially simplify genotyping workflows, minimize errors, and save labor, time, and cost.

## 1. Introduction

Sweet cherry (*Prunus avium* L.) produces one of the earliest ripening and most tasty fruits in temperate climates, making it a popular choice among consumers. It is therefore not surprising that worldwide production is constantly increasing, reaching nearly 3 million tons in 2023 (www.fao.org (accessed on 12 September 2025)). The three main producers are Turkey, Chile, and the USA, followed by Uzbekistan, Iran, Greece, and Spain. It is estimated that there are thousands of sweet cherry varieties in the world, and breeding programs are ongoing to develop new and better varieties with extended ripening periods. For instance, the U.S. Department of Agriculture alone has more than 800 sweet cherry entries on file (www.usda.gov (accessed on 12 September 2025)), and Schuster et al. [1] described 1700 different varieties.

For cherries in particular, it is extremely important to distinguish varieties with certainty, as they differ in critical parameters for successful cultivation. Some are self-compatible, enabling them to grow in monocultures, but most varieties are self-incompatible [1] and need a pollination partner. To cross-pollinate each other, they have to possess at least one different so-called S-allele, a variant of the locus responsible for controlling pollination success [1,2]. Furthermore, for successful harvesting, it is necessary to grow a variety in a given area that is capable of meeting its chilling hour requirements to end dormancy, ensuring ample blooming [2,3]. When dormancy is not released naturally, dormancy breakers such as Dormex^®^ have to be used, increasing the cost of production [4]. The time of flowering is another critical trait, as growing early-flowering varieties increases the risk of crop loss in areas with frequent spring frosts, and expensive anti-freezing measures must be used when temperatures drop below zero [5]. Finally, varieties differ in such parameters as fruit size, firmness, and color; trees may be more or less susceptible to biotic and abiotic stresses, or ripen in different periods. Selection of the right sweet cherry variety is, therefore, a critical step for a farmer’s success. Hence, it is not surprising that growers want to be sure about the variety they plant in newly established orchards that will be managed for many years.

The identification/verification of sweet cherry varieties is usually done by phenological and phenotypical evaluation. However, assessing fruit-bearing trees requires at least two years to minimize inter-seasonal variation, and the evaluation must be performed by highly experienced specialists. On the other hand, molecular genetic approaches offer much quicker and more reliable techniques for identifying varieties, use a minimal amount of biological material, and can be performed throughout the year, even in young seedlings or trees without fruits. This approach is called genotyping or fingerprinting, and uses molecular markers with sufficient discrimination power to distinguish individual varieties.

Nowadays, genotyping is commonly performed with SSR (Simple Sequence Repeat) or SNP (Single Nucleotide Polymorphism) markers. Both types of markers have their advantages and disadvantages. SSR markers, suitable for genotyping, should be highly polymorphic, independently inherited (no genetic linkage between them), and should exhibit low stuttering and consistent reproducibility [6]. Twelve or fewer SSR markers are usually sufficient to distinguish fruit crop varieties, including those capable of self-pollination. The number of SSR markers recommended for reliable variety differentiation depends on the marker informativeness, the genetic diversity of the fruit species [6], and ploidy (higher species ploidy may lower the number of SSR markers needed for genotyping [7,8,9]). On the other hand, SNPs are suitable for extensive genotyping projects and for the construction of genetic maps, lowering the cost per analysis. As SNPs are usually bi-allelic, it is necessary to analyze substantially more SNPs than SSR markers, which requires advanced biostatistic methods to interpret results [10]. Despite advances in whole-genome sequencing techniques, SSR markers remain the gold standard for genotyping not only in fruit crops but also in humans, as SNPs are less suitable and too expensive for routine genotyping of low numbers of samples [6].

Many SSR markers have been developed in sweet cherries [11,12,13], and SSRs from other closely related diploid *Prunus* species—such as peach [14,15,16], Japanese plum [17], and almond [18]—have also been used for their genotyping. In 2009, the European Cooperative Programme for Plant Genetic Resources (ECPGR) recommended 16 SSR markers for genotyping sweet cherries by fragment analysis [19]. These markers have been used for genotyping several collections, e.g., 383 unique genotypes from the German Fruit Genebank [20]; however, some authors have revised the ECPGR SSR marker set and added new markers as some varieties could not be distinguished [21,22].

For SSR marker analysis, a capillary genetic analyzer is usually used to separate PCR fragments with one-nucleotide accuracy. This method uses several different fluorescent dyes in parallel with amplicons ranging from 80–600 bp in length, allowing the analysis of several SSR markers in one reaction. This approach, familiar from human genetics, has also been adapted for fruit crop genotyping. For example, Chambers et al. [7] amplified and analyzed eight SSRs in a single reaction to genotype up to octaploid strawberries; Akin et al. [23] multiplexed 14 SSRs in one reaction to genotype diploid hazelnuts; Zurn et al. [24] designed primers to co-amplify 10 SSRs in one reaction to distinguish accessions in the United States National *Pyrus* collection (diploids and triploids); Bassil et al. [8] published a 10-SSR multiplex for fingerprinting up to octaploid blueberries; Čmejlová et al. [25] optimized a single reaction amplifying 17 SSRs for genotyping diploid and triploid apples; and Čmejlová et al. [9] developed a set of eight SSR markers for the identification of hexaploid plum varieties in one reaction.

Genotyping by SSR markers may not only be useful for confirming the identity of varieties, but can also be used for further analyses. If a sufficient number of SSR markers is analyzed in a sufficient number of varieties (the number of used varieties depends on SSR/population heterogeneity), it is possible to use SSRs for parentage, pedigree, and/or population analyses. For these purposes, highly polymorphic markers distributed across the genome are needed. Information about parents is highly valued by breeders, while pedigree and population analyses are important mainly for scientists exploring relationships and diversity, especially in gene pools. The usefulness of SSR markers for these analyses has been confirmed, for example, by Barreneche et al. [21], Marchese et al. [26], or Liu et al. [27] in population and pedigree analyses of sweet cherries; Liu et al. [28] developed SSR markers for paternal identification in *Camellia sinensis*; Zhang et al. [29] identified parents in walnut F1 progeny by SSRs; and Vuletin Selak et al. [30] used SSR markers to study paternity in seeds from mixed olive orchards.

The objectives of this work were (i) to identify new high-quality SSR markers using whole-genome sequences of 299 sweet cherry varieties or hybrid material; (ii) to develop an SSR marker set for genotyping sweet cherries with high discrimination power; (iii) to provide an easy and quick genotyping assay in one reaction; and (iv) to use the results obtained by the assay for advanced analyses (pedigree, population, and parentage analyses). This one-reaction approach should make the determination of sweet cherry varieties accessible not only to the scientific community but also to national regulatory authorities and the general public for routine analyses at relatively low cost. The final aim of this study was to compare the newly developed identification system with the previously used genotyping system recommended by the ECPGR.

## 2. Results

**Figure 1:**
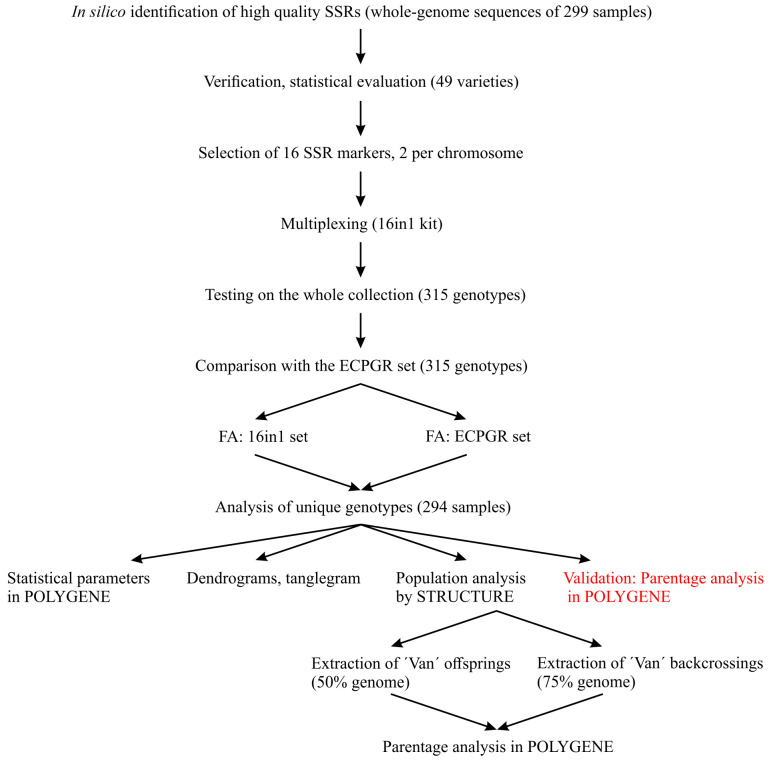
Workflow of the study. This study was conducted in four steps: (i) identification and selection of new SSR markers; (ii) multiplexing selected markers in one reaction; (iii) testing and comparison with ECPGR markers; and (iv) exemplary analyses. FA: Fragment analysis.

This study was conducted according to the workflow depicted in Figure 1 and is described in detail in the following sections. The list of varieties/genotypes used in this study is presented in Supplementary Table S1.

### 2.1. Newly Identified SSR Markers

Based on 299 whole-genome sequences, 30 new SSR markers were extracted from the sweet cherry genome of the ‘Tieton’ variety (Table 1) that passed the following criteria: minimally, three alleles with a maximum allele frequency of 0.5; relatively short repetition to avoid high stuttering, and polymorphic information contents based on allele sequences (PIC_HipSTR_) greater than 0.8 for SSR markers with dinucleotide repetition, 0.7 for markers with trinucleotide motif, and 0.6 for SSRs with longer repetition unit. Most of the identified SSRs possessed a dinucleotide repetition (19 SSRs), while five SSRs were characterized by a repetition composed of three or four nucleotides, and one SSR marker contained a hexanucleotide repetition. Some of these SSR markers were located in a gene, but in non-coding regions (introns, untranslated regions); most were situated in intergenic regions (Table 1).

All 30 SSRs were successfully verified by fragment analysis on 49 samples to allow statistical evaluation (Table 2; primers used for this analysis are shown in Supplementary Table S2). The number of alleles in individual markers ranged from 3 to 11, with the number of effective alleles (Ae) from 2.553 (Pav_chr7_863) to 6.651 (Pav_chr3_002). Although PIC_HipSTR_ was one of the criteria for selection of high-quality SSR markers, the observed PIC values based on fragment analysis in the chosen subpopulation of 49 varieties were lower for some SSR markers, ranging from 0.540 (Pav_chr7_863) to 0.833 (Pav_chr3_002). Lower PIC values from fragment analysis compared to PIC_HipSTR_ values were expected since the HipSTR algorithm works with the sequence of alleles, while fragment analysis discriminates only the length of alleles, irrespective of their sequence. The existence of a one-length allele with two different sequences was confirmed by the presence of SNPs in samples homozygous for the particular SSR marker in the fragment analysis (for example, Pav_chr2_995, Pav_chr6_387, Pav_6_989, or Pav_chr7_863). Expected heterozygosity varied between 0.608 (Pav_chr7_863) and 0.850 (Pav_chr3_002); the minimal observed heterozygosity of 0.571 was found in Pav_chr7_863, and the maximal observed heterozygosity of 0.918 was detected in Pav_chr3_706.

### 2.2. Multiplexing of 16 SSR Markers into One Reaction

**Figure 2:**
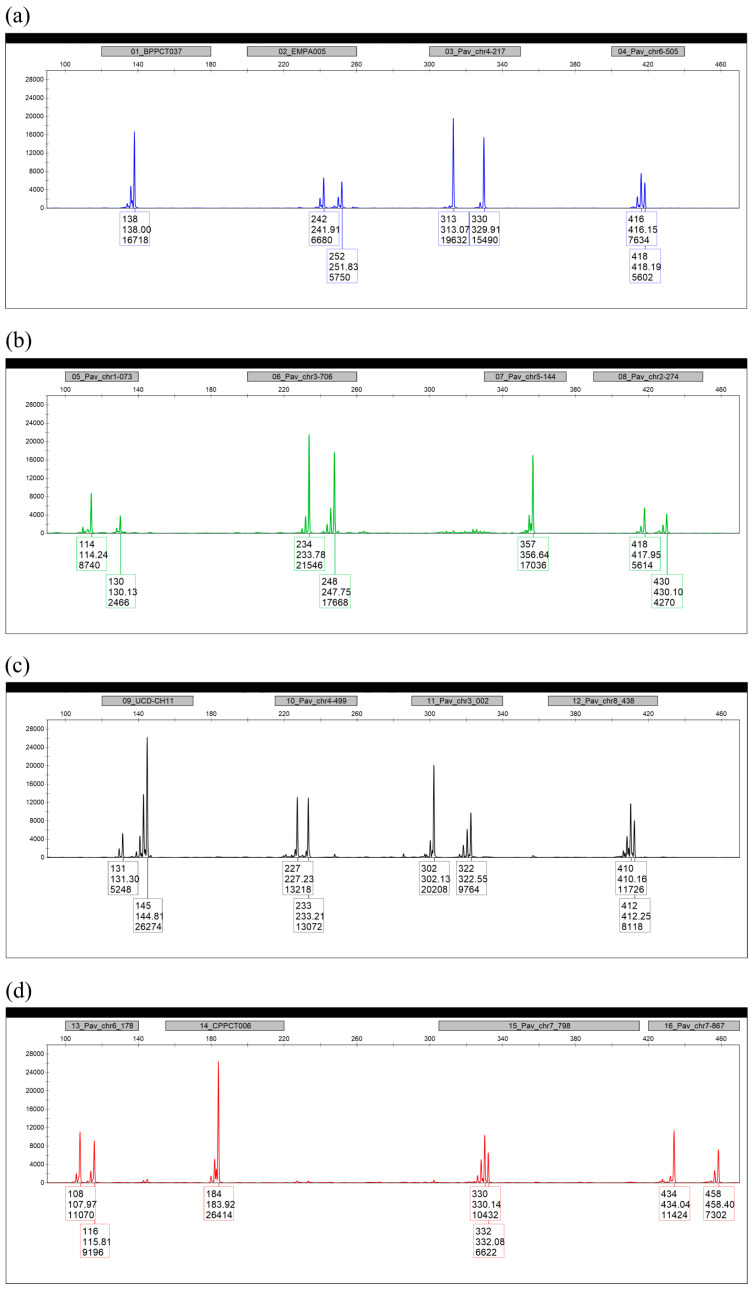
Representative output of fragment analysis in the ‘Van’ variety using the 16in1 genotyping system. All channels have the same scales. (**a**) Blue detection channel (6-FAM label): BPPCT037, EMPA005, Pav_chr4_217, and Pav_chr6_505 marker; (**b**) green detection channel (VIC label): Pav_chr1_073, Pav_chr3_706, Pav_chr5_144, and Pav_chr2_274; (**c**) yellow detection channel (NED label): UCD-CH11, Pav_chr4_499, Pav_chr3_002, and Pav_chr8_438; (**d**) red channel (PET label): Pav_chr6_178, CPPCT006, Pav_chr7_798, and Pav_chr7_867.

Two SSR markers per chromosome with the highest Ho, He, PIC, and Ae values were chosen for multiplexing into one PCR (Table 2). Positions of the SSR markers were also taken into account since two SSR markers identified on chromosome 2 were separated by only a 200 kbp region, and on chromosome 5, Pav_chr5_144 and Pav_chr5_903 lay only 70 kbp from each other. However, SSR markers Pav_chr1_028, Pav_chr5_059, Pav_chr6_641, Pav_chr7_286, and Pav_chr8_224 failed to amplify in a multiplex setup, even if very high concentrations of primers were used. Therefore, alternative SSR markers extracted from previously known genetic maps were also tested, and the best ones from each chromosome were chosen: EMPA005 (chr1), UCD-CH11 (chr2), BPPCT037 (chr5; ECPGR recommended), and CPPCT006 (chr8; ECPGR recommended). The main criteria for their selection were high polymorphism, specificity under the used PCR conditions, and low stuttering. Thirty-two primers for PCR amplification in one reaction were successfully multiplexed, and concentrations of primers were fine-tuned to obtain comparable signal heights for all markers. Alleles with a very low signal were observed for UCD-CH11. Whole-genome sequences in the respective samples were checked, and an A/C polymorphism in the forward primer-annealing site was found. To improve annealing, the primer was degenerated in this position, and while the signal of allele 131 remained somewhat lower, it was sufficient for reliable analysis. Primers for the amplification of the final set of 16 selected SSR markers, hereafter termed the 16in1 system, are summarized in Table 3. Figure 2 demonstrates a typical output from fragment analysis on a capillary genetic analyzer.

### 2.3. Statistical Evaluation

The 16in1 genotyping system was verified on 315 samples (most of them analyzed in multiple biological replicates); allele combinations for each marker in each sample are summarized in Supplementary Table S3. During testing, clones of one variety (Supplementary Table S3, differing alleles are background colored), and several misidentifications and/or synonyms that showed the same genotypes but different names were identified (Supplementary Table S4). These discrepancies were also confirmed using the ECPGR marker set (Supplementary Table S5). On the other hand, samples identified as identical by the ECPGR system were separated by the 16in1 kit into two or even three varieties, differing by only one or two alleles (Supplementary Table S6). This means that these samples had identical genotypes or differed by only one or two alleles in 30 analyzed SSR markers (two SSR markers are common for both 16 SSR marker sets); therefore, they were labeled herein as varieties/clones. To statistically evaluate the quality of individual SSR markers and the whole set, only unique 16in1 genotypes/varieties were used (Supplementary Table S3).

The results of 294 unique genotypes using the 16in1 genotyping system were statistically evaluated in POLYGENE software (version 1.7) (Table 4). The highest polymorphism was observed for SSR marker Pav_chr3_002 (PIC 0.846; Ae 7.223), and the lowest for EMPA005 (PIC 0.606; Ae 2.832). The whole 16in1 genotyping system has an average PIC of 0.767 with Ae of 5.227. Additional statistical parameters, including the allelic richness, number of private alleles, and Shannon’s information index, can be found in Supplementary Table S7. The probability of the random identity of two genetically different samples for the whole set of SSR markers (PI_t_) was calculated to be 1.27 × 10^−19^. All markers were amplified in all samples tested; no null alleles were recorded (the probability of null alleles ranged from 0.011 to -0.035). Statistical parameters of the collection of 49 cultivars were compared with those of the full set of 294 cultivars, and the PIC values for individual markers differed by no more than 5%. The cultivar collection used for the primary analyses therefore provided a reasonably good representation of all analyzed cultivars). An overview of all alleles with their frequencies is presented in Supplementary Table S8.

For comparison, the same set of unique genotypes was also analyzed with the ECPGR SSR marker set. The statistical evaluation of results obtained by ECPGR markers (Table 5) showed that EMPAS06 was the best SSR marker with PIC 0.806 and Ae 5.813; however, EMPA017 had the lowest PIC value of 0.235 and Ae of only 1.329. Other statistical characteristics are summarized in Supplementary Table S9. Varieties with homozygous null alleles (i.e., no amplification of this marker in some samples) were observed in SSR markers EMPaS10, EMPaS06, and EMPA026 (Supplementary Table S10; also contains a list of all identified alleles and their frequencies). The whole ECPGR genotyping system showed an average PIC of only 0.564 and Ae of 2.994, and the PI_t_ value for the analyzed samples was calculated to be 1.00 × 10^−12^.

### 2.4. Population Analysis

**Figure 3:**
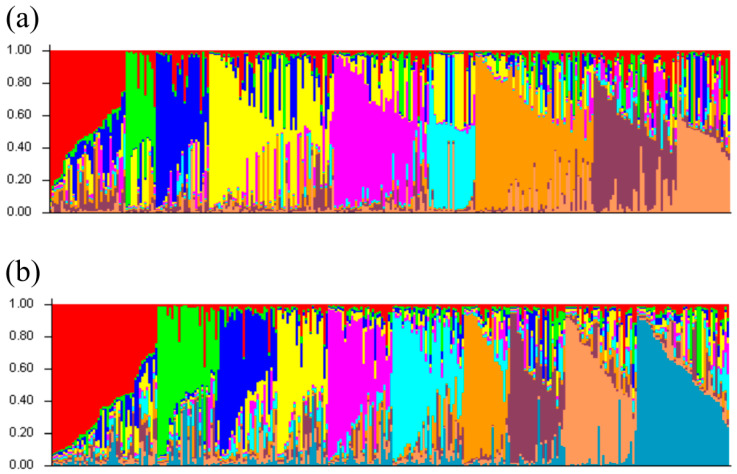
STRUCTURE analysis of 294 unique genotypes. (**a**) Using the 16in1 system, the population was divided into nine groups (K9), while (**b**) the use of the ECPGR system resulted in ten groups (K10).

The results obtained with both SSR marker sets were further used for population analysis in the STRUCTURE software (version 2.2.4). Dendrograms for both 16in1 and ECPGR marker sets were created in POLYGENE to establish the rank of samples for their input to STRUCTURE (Supplementary Table S11). The analysis divided the 16in1 analyzed samples into 3 and 9 groups with the highest delta Ks of 189.02 and 37.11, respectively, while the ECPGR analyzed samples were separated into 2 and 10 groups with the highest delta Ks of 58.82 and 45.39, respectively (Supplementary Table S12). The composition of individual groups was evaluated from a pedigree perspective and considering geographical origin (K3 and K2 groups). ‘Bing’, ‘Germersdorfer’, ‘Kordia’, ‘Lambert’, ‘Rube’, ‘Schmidt’, ‘Stella’, and ‘Van’ were repeatedly observed in pedigrees of varieties with high Score–Inferred clusters (S-Ic), and the frequency of their incidence in groups was therefore evaluated. For the finer subdivision into groups (K9 and K10), only those representatives with an S-Ic > 0.9 were highlighted, as some groups contained only a small number of varieties. Figure 3 depicts the results of STRUCTURE analysis for both 16in1 and ECPGR marker sets sorted by Q.

For further analyses, the STRUCTURE groups resulting from the 16in1 system were labeled K3.1 to K3.3, and K9.1 to K9.9 (Supplementary Tables S13 and S14). The K3.1 group contained 58 samples in total; 29 varieties had an S-Ic higher than or equal to 0.8. In this group, modern cultivars prevailed—25 out of 29 varieties with the highest S-Ic had ‘Van’ (18/29), ‘Stella’ (10/29), ‘Kordia’ (7/29), or ‘Bing’ (5/29) in its pedigree, while 4/29 had an unknown origin. From a geographical point of view, 13 out of 29 varieties originated in Canada, and 9/29 were bred in the Czech Republic. The K3.2 group was composed of 117 highly heterogeneous samples, many of which have an unknown pedigree and may be considered landraces. Sixty-five of them had an S-Ic higher than 0.8. Six varieties out of twenty with known parents, and an S-Ic ≥ 0.8 had ‘Germersdorfer’ in their pedigree, while the ancestor of four varieties was ‘Schmidt’. Geographically, 17/65 varieties originated from Germany, and 10/65 from the USA. The remaining 119 varieties belonged to the K3.3 group. This group was also very heterogeneous, and contained many varieties with unknown pedigree and landraces. Sixty varieties had an S-Ic higher than 0.8; ‘Rube’ was one of the parents in 7/23 varieties with a known pedigree in this subgroup. Varieties coming from Europe clearly prevailed—only 4 varieties out of 60 with an S-Ic ≥ 0.8 had their origin outside of Europe (USA); the highest number of varieties from one country had their origin in Germany (17/60 varieties).

At a finer K9 distribution, the highest S-Ics (>0.9) in individual groups were found for ‘Droganova’, ‘Germersdorfer’, ‘Boambe de Cotnari’ (a clone of ‘Napoleonova’), ‘Hedelfingenska’, ‘Rebekka’, ‘Lyonska rana’, ‘Van’, and ‘Nero II c1’. ‘Reidler’ had the best score in group K9.8, but only 0.848. In the 16in1 genotyping system, K9.4 (‘Hedelfingenska’ group) and K9.7 (‘Van’ group) prevailed, with 54 and 51 members, respectively. Many identified representatives of individual groups corresponded to varieties frequently used in crossings, e.g., ‘Germersdorfer’, ‘Van’, or ‘Nero II c1’ (Supplementary Table S2).

Similarly, groups analyzed by the ECPGR system were labeled K2.1 to K2.2, and K10.1 to K10.10 (Supplementary Tables S15 and S16). K2.1 contained 152 varieties; 110 of them showed an S-Ic higher than or equal to 0.8; modern cultivars with a known pedigree predominated. As for geographical origin, 27 of them came from Canada, 21 originated from Germany, 19 were registered in the Czech Republic, and 13 of them were found/bred in the USA. K2.2 was represented by 142 varieties; 93 of them had an S-Ic ≥ 0.8. European landraces represented the main part of this group. Thirty of them came from Germany, and twelve from the Czech Republic. The representatives of individual groups for K10 with S-Ic scores >0.9 were ‘Van’, ‘Lyonska rana’, ‘Nero II c1’, ‘Alfa’, ‘Cristimar’, ‘Rebekka’, ‘Napoleonova’, ‘Zukunft’, and ‘Mednanska’. In group K10.4, two varieties (‘Germersdorfer’ and ‘Schneiders Spaete Knorpelkirsche’) sharing the same ECPGR genotype were identified (score 0.895). The largest ECPGR groups were K10.1, comprising 46 varieties (the ‘Van’ group), and K10.10 (‘Mednanska’) with 40 genotypes. Identified representatives of individual groups corresponded to varieties frequently used in crossings (Supplementary Table S2).

To summarize the results of the K3 (16in1) and K2 (ECPGR) group analyses, the 16in1 genotyping system resulted in a clearer distribution of genotypes. Most of the modern cultivars were grouped into K3.1; K3.2 and K3.3 were heterogeneous with a high content of landraces. Non-Europe genotypes concentrated in K3.2, whereas K3.3 contained only a small number of them. Varieties with ‘Germersdorfer’ and ‘Schmidt’ in their pedigree were assigned to K3.2; known relatives of ‘Rube’ were only found in group K3.3. In contrast, the ECPGR groups were less clearly defined from both pedigree and geographical origin points of view; however, modern cultivars were preferentially found in the K2.1 group.

Comparing representatives of individual groups K9 and K10 obtained by the 16in1 and ECPGR systems, respectively, six groups exhibited the same best representative (‘Germersdorfer’, ‘Boambe de Cotnari’ (clone of ‘Napoleonova’), ‘Rebekka’, ‘Lyonska rana’, ‘Van’, and ‘Nero II c1’). Moreover, according to the STRUCTURE results and dendrograms, ‘Hedelfingenska’ as a representative of K9.4 is closely related to ‘Mednanska’ (K10.10). The size of related groups was similar, e.g., K9.3 and K10.8, both represented by ‘Napoleonova’, contained 23 and 24 varieties, respectively; but some groups differed substantially, for example, group K9.5 of ‘Rebekka’ consisted of 41 varieties, while corresponding K10.7 comprised 20 varieties.

Next, a tanglegram comparing dendrograms generated from the results of both the 16in1 and ECPGR systems was constructed to further evaluate similarities between the genotyping systems (Supplementary Table S17). Dendrograms were rearranged with respect to each other, and although some similarity between sample groups may be observed, only several varieties were distributed in exactly the same subtrees (branches) in both dendrograms (highlighted by colored lines between individuals in the two dendrograms). The tanglegram also shows that several varieties genotyped by ECPGR-recommended SSR markers having the same genotype were distinguished by 16in1 SSR markers, but by only one or two differing alleles. The tanglegram Mantel’s statistic value of r = 0.5552 indicated a moderately strong positive correlation between both dendrograms, and the genetic distances between individuals were similar in both sets of markers (*p*-value 0.001).

### 2.5. 16in1 Kit Validation: Parentage Analysis

Parentage analysis in POLYGENE software was used to look for parent pairs for all samples, using a very strict criterion of null Trio loci mismatching (Supplementary Table S18). Some suggested parent combinations were pairs, including genotypes differing by only one allele (for example, ‘Napoleonova’, ‘Boambe de Cotnari’, and ‘Esperens Knorpelkirsche’); these were counted only once for statistical evaluation (Supplementary Table S19). Suggested parents were compared with the parent combination for the particular variety, if known.

For the 16in1 system, 244 parent pairs were discerned for 294 analyzed samples. After removing parent pairs containing clonal varieties, 189 parent pairs remained. At least one parent combination without mismatching alleles was found for 123 out of 294 samples, i.e., 1.54 parent pairs were identified per variety on average. The whole collection contained 34 varieties with both parents known and analyzed, and 28 (82.35%) were successfully identified as the only one suggested parent combination.

In comparison, one to five parent combinations were suggested in 190 varieties using the ECPGR set of SSR markers. In total, 654 parent pairs were chosen by POLYGENE; after excluding parent pairs consisting of clones, 521 parent combinations remained, i.e., 2.74 parent pairs without mismatching alleles were found per variety on average (however, only the top five LOD score pairs with a null score of Trio loci mismatching were extracted). The correct parent combination, as the unique one, was identified in only 5 out of 34 varieties (14.71%).

**Figure 4:**
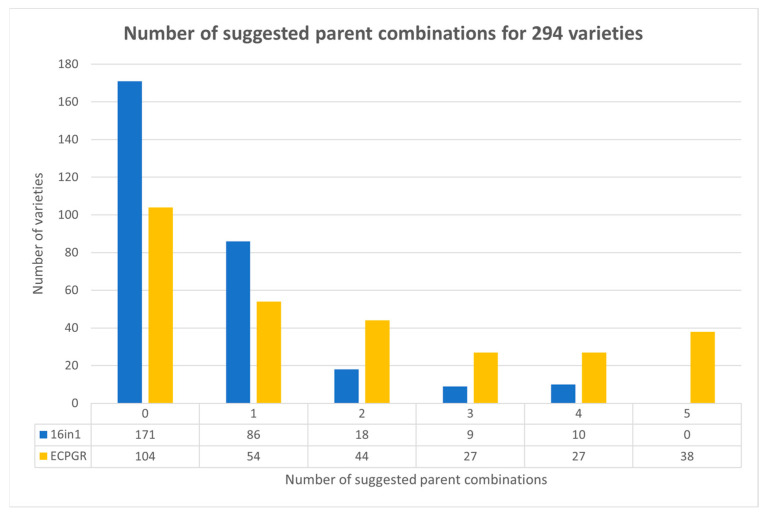
Comparison of parentage analysis based on the 16in1 and ECPGR systems. “0” indicates no parent combination was identified. The table under the graph indicates the number of varieties with the specified number of predicted parent combinations. Using the 16in1 system, for 123 varieties, one to four parent combinations were found. For the ECPGR marker set, one to five parent combinations were suggested in 190 varieties.

Using the 16in1 SSR marker set, a single parental combination was proposed for 86/123 (69.9%) varieties for which a parental combination was suggested, and no variety had five parent pairs identified. By contrast, using the ECPGR markers, a unique parental combination was proposed for only 54/190 (28.4%) varieties, and 38/190 (20%) of varieties had five suggested parent pairs. The number of identified parent pairs for all analyzed samples is summarized in Figure 4.

Combining the results of 16in1 and ECPGR analyses, the same parent pairs were suggested for 54 varieties with unknown parents or open-pollinated mothers (Supplementary Table S19). For example, ‘Grace Star’ came from a crossing of ‘Lapins’ and ‘Burlat’; for ‘Horka’, ‘Van’ was identified as a pollinator of ‘Kordia’; parents of ‘Starking Hardy Giant’ were identified as ‘Windsor’ and ‘Schmidt’; and ‘Star’ was an offspring of ‘Deacon’ and ‘Lambert’.

### 2.6. Analysis of ‘Van’ Relatives: An Example of a Highly Inbred Group

The group of ‘Van’ relatives presents the most challenging task for cherry genotyping and parentage analysis using SSR markers because of a very high degree of inbreeding. Using the 16in1 system, the ‘Van’ family was defined as those from the K9.7 group based on STRUCTURE analysis that contained more than 50% of the K9.7 genome (Supplementary Table S20). This group, named K9.7-50%, contained 35 varieties; most of the members were known offspring of ‘Van’; however, some accessions had no known parentage. In the next step, the group was narrowed to those with 75% of the K9.7 genome; this K9.7-75% group comprised 14 varieties. A strong drop in heterozygosity was observed, and the PIC value decreased from 0.767 (whole collection) to 0.597 (K9.7-50%) and 0.506 (K9.7-75%), respectively. Concomitantly, Ae declined from 5.227 to 3.136 and 2.557, respectively (Table 6). Loss of alleles in all individual SSR markers was detected and may be found in Supplementary Table S20. Hence, the original PI_t_ decreased from 1.27 × 10^−19^ to 2.37 × 10^−13^ (K9.7-50%) and 1.04 × 10^−10^ (K9.7-75%), respectively.

The ECPGR system also showed a high decline in these parameters (Table 7, more details in Supplementary Table S21)—the PIC value calculated for all samples (0.564) dropped to 0.402 for K10.1-50% (the group of ‘Van’ relatives in the ECPGR system; 36 varieties) and 0.356 in the case of K10.1-75% (18 genotypes), and the original PI_t_ value decreased from 1.00 × 10^−12^ to 1.86 × 10^−8^ (K10.1-50%) and 1.27 × 10^−7^ (K10.1-75%). Loss of alleles in individual SSR markers may be found in Supplementary Table S21.

## 3. Discussion

Genotyping of sweet cherries is a challenging task due to low heterogeneity, especially in modern varieties, which stems from the tendency to breed self-compatible varieties that are usually derived from ‘Stella’ and its descendants [1], and/or the repeated usage of a limited number of self-incompatible varieties for breeding. For example, Choi and Kappel [31] analyzed inbreeding and founding clones for sweet cherries coming from North America, and 64 out of 66 analyzed modern varieties were found to be descendants of only five founding clones (‘Black Heart’, ‘Emperor Francis’, ‘Empress Eugenie’, ‘Napoleon’, and ‘Windsor’). A loss of heterozygosity in modern varieties was also confirmed by Campoy et al. [32], and a narrow genetic bottleneck in modern cherry varieties was already reported in 2010 [33]. The problematic differentiation of closely related varieties was also shown by the work of Wiersma et al. [34].

Both SNP and SSR markers are commonly utilized for fingerprinting plant varieties. Modern techniques frequently focus on SNP markers, and methods such as genotyping-by-sequencing and SNP chips are commonly used for their detection. Forty SNP markers screened by high-resolution melting analysis were successfully used for genotyping 143 varieties of sweet cherries by Fernandez i Marti et al. [35]. Campoy et al. [32] performed the first population genetic analysis in cultivated sweet cherries using a medium-density SNP marker array, RosBREED cherry 6 K SNP array v1, on 210 genotypes. Recently, Palasciano et al. [36] obtained 13,117 SNPs through the genotyping-by-sequencing of 143 cherry varieties, and proved that SNPs are an excellent tool for assessing genetic diversity and relationships. However, as demonstrated by Testolin et al. [6] and others, while SNPs are the markers of choice for en masse genotyping and in-depth analysis of genomes, close-kin differentiation or for constructing genetic maps, especially in large collections, SSR markers are still prioritized for routine genotyping. SSR marker analysis is relatively cheap, rapid, reliable, and simple to interpret, and using appropriate controls, the method can easily be implemented in other laboratories. A capillary genetic analyzer needed to perform fragment analysis is also available in many labs. Moreover, analysis of tens of SSR markers usually provides sufficient information for identifying varieties, i.e., only “small data” are produced that can be easily handled and shared. On the other hand, SNP genotyping is a modern trend in genetics that generates “big data” requiring a skilled bioinformatician for interpretation. In addition, expensive laboratory equipment is needed, so this type of analysis is usually outsourced, and laboratories thus lose full control over the analytical process. As there are many protocols, workflows, and pipelines for genotyping-by-sequencing, the reproducibility of results between labs may be of concern. The RosBREED cherry 6 K SNP array v1 from Thermo Fisher Scientific may represent a standardized genotyping platform, but it is no longer available. Moreover, Wiersma et al. [34] confirmed the difficulty of distinguishing closely related cultivars by both SSR and SNP markers. Unsurprisingly, informative SNP markers were located in the same regions as discriminating SSR markers. In light of these findings, the development of a cheap, fast, and easy-to-use genotyping tool based on SSRs seems ideal for routine sweet cherry genotyping.

The ECPGR recommended set of 16 SSRs [19] has long been used for cherry genotyping. However, in our study, some of these markers had very low polymorphism: 10 out of 16 SSR markers showed PIC values below 0.6, with the number of effective alleles <3. The average values for the whole ECPGR system were also rather low, with PIC of 0.564 and Ae of 3.0. It is not, therefore, surprising that some authors have tried to improve the ECPGR set [21,22]. In probably the largest genetic diversity and structure analysis of a combined sweet cherry germplasm from across Europe, Barreneche et al. [21] analyzed 314 cherry varieties obtained from 19 countries. Most of the collection represented original landraces from the country of provenance; the rest were popular old and modern varieties. Their final set of 14 SSR markers used for genotyping included nine loci from the ECPGR recommended genotyping set, two newly selected SSR markers previously used for cherry genotyping by different scientific groups, two loci associated with fruit weight/size and broadly used for molecular marker-assisted selection in breeding (BPPCT034 and CPSCT038), and one SSR marker genetically linked to fruit color (Pav-Rf-SSR) (reviewed in [37]). The PIC values of this system were 0.661 for 111 landraces and 0.639 for 104 early selections together with modern breeding varieties. It is noteworthy that among 314 varieties, 40 groups with shared genotypes were found. Some new supposed redundancies were identified, and others were confirmed; however, mislabeling and synonyms were not sufficient to explain all redundancies [21]. Furthermore, SSR markers that are widely utilized for the molecular-marker-assisted selection of seedlings with the perspective of bearing large fruit or having certain fruit color are not suitable for genotyping of modern varieties because they tend to be homozygous.

As only a limited number of highly polymorphic SSR markers suitable for the genotyping of sweet cherries have been described, we decided to identify new markers using the whole-genome sequences of 299 unique genotypes [38]. Using strict criteria, 30 SSR markers from all chromosomes were identified; most of them (20/30) were located in an intergenic region, the rest (10/30) were in non-coding sequences of predicted proteins (introns, 3’ untranslated regions). Some of the developed SSRs failed to be amplified in a multiplex PCR despite working in a simplex PCR setup. All of these contained AT-rich repetitions, underlying a poor rate of successful PCR amplification, as has also been shown for other SSRs, for example, in rice [39]. Other SSR markers were excluded due to their lower heterogeneity or close proximity to a more polymorphic SSR marker. A distance of at least 1 Mbp between SSR markers was chosen as the threshold to minimize the probability that markers showed genetic linkage. The selected distance was tenfold higher than the average distance at which intra chromosomal linkage disequilibrium (LD) declined below r^2^ = 0.2 [32], i.e. the threshold below which LD is considered low or negligible. Finally, twelve newly identified SSR markers were multiplexed with four previously known SSRs showing high polymorphism, in such a way that two SSR markers represented each of the eight *Prunus avium* chromosomes.

The final 16in1 genotyping set was optimized to be used in one PCR and fragment analysis reaction. It has an average PIC value of 0.767, which is higher than the PIC values of both the ECPGR system (tested on the same collection) and the set developed by Barreneche et al. [21]. The total probability of the random identity of two genetically different samples, PI_t_, was 1.27 × 10^−19^, which is seven orders of magnitude lower than the PI_t_ for the ECPGR set (1.00 × 10^−12^). This value reflects the lower heterogeneity of sweet cherries in comparison with apples and pears, where much lower values were obtained with 17 SSR markers, though based on a higher number of genotypes: PI_t_ ~ 10^−22^ for apples (880 genotypes; [25]), and PI_t_ ~ 10^−25^ for pears (322 diploid genotypes; manuscript in preparation). However, it is necessary to keep in mind that the PI_t_ value was obtained using a collection of European and North American varieties; results may differ for other collections or geographical regions, e.g., in Asia or in wild cherries.

Our newly developed 16in1 system was compared with the currently used ECPGR system, which contains the same number of SSR markers. Though both SSR sets worked similarly in genotyping tasks, their performance differed in population analyses and, in particular, in parentage assessments. The 16in1 set showed a more precise distribution into STRUCTURE groups, and critically led to an improvement in the identification of parent pairs. Whereas the ECPGR set of SSR markers provided many possible parent pairs for individual varieties, the 16in1 set usually suggested only one parent combination per variety and correctly identified the right parents in more than 82% of varieties in which both parents are known.

One of the most challenging tasks for SSR genotyping is the discrimination of genotypes within highly inbred groups with the possible existence of breeding bottlenecks and founder effects [33]. To better test our 16in1 system, cherries from the ‘Van’ family were chosen, since these varieties are repeatedly crossed with each other, and the ‘Van’ groups detected by STRUCTURE in both genotyping systems contained a sufficient number of varieties for further analysis (16in1: K9.7 consisted of 51 genotypes; ECPGR: K10.1 contained 46 varieties). As expected, losses of alleles, along with reductions in the number of effective alleles and in average PIC values, were observed, suggesting the presence of a breeding bottleneck and a founder effect in this group. However, the main goal of the ‘Van’ group analysis was to test the reduction rate of discrimination power in such a highly inbred group. When analyzed by the new 16in1 set of markers, PIC dropped from 0.767 for the whole collection to 0.597 for varieties with half of their genome corresponding to the 16in1 K9.7 group, and 0.506 for varieties whose genome harbors 75% of the K9.7 group. However, these values remain similar to the PIC values achieved by the ECPGR system across the entire collection of varieties, highlighting the improved discriminatory power of the 16in1 system. For comparison, the ECPGR system showed a decline in PIC to 0.402 for K10.1-50% and to only 0.356 for K10.1-75%.

In addition to the higher resolution capability of the 16in1 system, its formulation in a one-reaction assay makes it ideal for routine cherry genotyping since it reduces labor, time, cost per analysis, and mistakes. One-reaction cherry genotyping may now be readily available and affordable not only for scientific purposes, but also for professional cherry growers, nursery managers, cherry breeders, germplasm curators, or hobby gardeners.

## 4. Materials and Methods

### 4.1. Plant Material

A total of 235 sweet cherry accessions from the Collection of Genetic Resources of the Research and Breeding Institute of Pomology Holovousy Ltd. (RBIPH) (Holovousy, Czech Republic) and 64 high-quality breeding plant materials were used for the identification of SSR markers. These genotypes and population structure are described in Holušová et al. [38]. Principal component analysis and kinship analysis revealed no subpopulations or strong sample clustering.

For SSR marker assessments, 315 genotypes were obtained from RBIPH, the Central Institute for Supervising and Testing in Agriculture, and the Fytos company (Plzeň, Czech Republic) (Supplementary Table S1). Accessions included commercial and older historic varieties, important breeding accessions, and traditional Czech landraces. If possible, accessions were analyzed at least in biological duplicates.

### 4.2. DNA Extraction

Genomic DNA for all analyses was extracted from 100 mg of shoot phloem using an Exgene Plant SV kit (GeneAll Biotechnology, Seoul, Republic of Korea) following the manufacturer’s protocol. To obtain a fine phloem powder, bark was removed from 1- to 2-year-old shoots with a scalpel, phloem was scraped off, and the tissue was ground to a powder in liquid nitrogen with a mortar and pestle. DNA quality was evaluated by a NanoDrop Lite Spectrophotometer (Thermo Fisher Scientific, Waltham, MA, USA), and DNA was diluted to 10 ng/µL for further analysis.

### 4.3. Simple Sequence Repeat Identification

Whole-genome sequences were retrieved from the Sequence Read Archive study number PRJNA813711 [38]. Genomic variants of microsatellites identified with MISA (MIcroSAtellite Identification Tool, version 1.0) [40] from the ‘Tieton’ Genome v2.0 reference sequence [41] were detected using HipSTR (Haplotype inference and phasing for Short Tandem Repeats) [42], which determined sequence variants for individual cherry varieties (299 samples). The criteria for selecting high-quality SSR markers were as follows:

1. The sequence failed to map, at most, 2 out of 299 whole-genome sequenced samples;
2. At least three alleles exist;
3. A maximum repeat length of 60 nucleotides to limit stutter;
4. A maximum allele frequency of 0.5 for any allele;
5. A polymorphic information content (PIC_HipSTR_) of 0.8 or higher for SSR markers with a dinucleotide repeat unit, 0.7 for SSRs with a trinucleotide repetition, and 0.6 for microsatellites with a longer repeat unit.

### 4.4. Fragment Analysis

Primers were designed using Primer3 software (version 2.6.1) [43]. Based on the sequence identity with the ‘Tieton’ v2.0, ‘Satonishiki’ v1.0.a1, and ‘Regina’ v1.0 reference genomes (https://www.rosaceae.org/organism/24337, accessed on 2 May 2024), PCR product specificity was determined using the in_silico_PCR.pl script (https://github.com/egonozer/in_silico_pcr, accessed on 2 May 2024). Primers producing nonspecific or duplicate amplicons were discarded. Reads from all varieties were mapped to the ‘Tieton’ v2.0 reference, and primers leading to amplicons that contained large insertions or deletions substantially affecting a PCR product length were discarded.

Fragment analysis for all 30 identified SSR markers was performed in 49 varieties (Supplementary Table S1). Cultivars were chosen on both the parental combinations (aiming for the widest possible range of parents) and the inclusion of landraces and cultivars of unknown origin from various parts of the world (Belgium, Bulgaria, the Czech Republic, Italy, Moldova, Germany, Russia, Slovakia, and the USA). Statistical parameters of the collection of 49 cultivars were compared with those of the full set of 294 cultivars, and the PIC values for individual markers differed by no more than 5%. The cultivar collection used for the primary analyses therefore provided a reasonably good representation of all analyzed cultivars. PCR protocol [9] was as follows: 5 μL of Phusion Flash High-Fidelity PCR Master Mix (Thermo Fisher Scientific), 1 μL of each primer (final concentration 0.2 µM, one of them fluorescently labeled by 6-FAM, Supplementary Table S2), 2 μL DNA (10 ng/μL), and PCR grade water up to 10 μL. PCR was run on a C1000 PCR cycler (Bio-Rad, Hercules, CA, USA) with the following temperature profile: 98 °C/1 min; 23 cycles (98 °C/10 s, 60 °C/15 s, 72 °C/15 s); final extension 72 °C/30 s. Afterward, 1 μL of PCR product was mixed with 15 μL Hi-Di Formamide (Thermo Fisher Scientific) and 0.5 μL GeneScan 600 LIZ dye Size Standard v2.0 (Thermo Fisher Scientific). Samples were denatured at 95 °C for 2 min and run on a 3500 Genetic Analyzer (Thermo Fisher Scientific). Results were analyzed in v5 GeneMapper software (Thermo Fisher Scientific).”

To verify PCR specificity, all PCR fragments were sequenced. Amplicons were separated by gel electrophoresis, corresponding fragments were cut out, and they were purified by a GeneAll Expin Combo GP kit (GeneAll Biotechnology). Fragments were sequenced using a BigDye™ Terminator v3.1 Cycle Sequencing Kit (Thermo Fisher Scientific) according to the manufacturer’s instructions. Sequences were analyzed in Geneious Prime^®^ software (version 2025.0; GraphPad Software LLC d.b.a Geneious, Boston, MA, USA).

### 4.5. Statistical Analysis

In all analyzed populations/subpopulations, POLYGENE software, version V1.7 [44] was used to investigate general SSR marker statistical parameters, such as observed heterozygosity (Ho), expected heterozygosity (He), number of effective alleles (Ae), number of alleles (k), or polymorphic information content (PIC). In the case of PIC, two types were calculated: PIC_HipSTR_, which was calculated based on alleles differing by their sequence according to the in silico analysis of whole-genome sequences, and standard PIC, which was derived from fragment analysis and was based on the length of individual alleles. As such, PIC_HipSTR_ was expected to be the same or higher than PIC in cases that one allele is represented by more sequences with the same length (e.g., due to an SNP presence). Lengths of the shortest and longest alleles were determined for all markers. For final statistical analysis, only the 294 genotypes that were unique according to our new system were used; if two or more genotypes for samples with the same name were observed, only the prevailing genotype was used for statistical evaluation. In cases of only two available samples, one of the clones was randomly chosen. The probability of identity (PI_i_) was calculated according to the formula given in [45]:

(1) $$PIi=\;\sum_{i}p_{i}^{4}+\;\sum_{i}\sum_{j>i}{\left(2p_{i}p_{j}\right)}^{2}$$

where *p_i_* and *p_j_* are population allele proportions. The total probability of identity (PI_t_) over the 16 SSR markers used was computed as the product of the PI of each individual marker.

### 4.6. Development of the 16in1 SSR Marker Kit

To construct the final kit, two SSR markers per chromosome, separated by a minimally 1 Mbp region, were chosen based on statistical parameters and multiplexed into one PCR reaction. Primers with four fluorescent dyes (6-FAM, VIC, NED, and PET) were used for the PCR amplification of fragments with approximate expected lengths 100–150, 200–250, 300–350, and 400–450 nucleotides to allow unequivocal assigning of all alleles to individual markers. The multiplex PCR reaction was run under the same conditions as the simplex one, but with 25 PCR cycles. Due to the inefficient PCR amplification of some SSR markers under multiplex conditions, and in cases where no alternative high-quality SSR marker was identified in this study, a known SSR marker was used instead. The following SSR markers extracted from genetic maps on www.rosaceae.org (accessed on 30 April 2024) were tested using original primers: Chromosome 1: EMPA002, EMPA003, EMPA005, UCD-CH31, UDP98-022, Pchcms4; Chromosome 2: Pchgms1, UCD-CH11, UCD-CH12, UDAp-461; Chromosome 5: BPPCT037 (the only one tested due to very high Ho, He and PIC in [24]); Chromosome 8: CPDCT034, CPPCT006, EMPA018, PS01H03. The best SSR markers from each chromosome were chosen for the final multiplex based on good PCR amplifiability, high polymorphic content, and easily scoring of fragment analysis outputs. The final set of 16 SSR markers obtained is referred to as the 16in1 set. Concentrations of primers were fine-tuned to obtain comparable signal heights for all markers. If alleles with a low signal were identified, the corresponding primer-annealing regions were checked in whole-genome sequences, and primers were appropriately modified. The final kit was tested on the whole collection containing 315 samples to obtain detailed statistics for each marker. All varieties were tested at least twice, preferably from different sources. Finally, all alleles were verified as belonging to a particular marker by a simplex PCR and fragment analysis.

For comparison, the ECPGR recommended set of SSR markers for sweet cherry genotyping was analyzed as described by Clarke and Tobutt [19]. All SSR analyses were run at least in duplicate.

### 4.7. Population Analysis

POLYGENE software, version V1.7 [44], was used to analyze relationships in a collection containing only unique genotypes according to the 16in1 set (294 samples). Hierarchical clustering was performed using the unweighted pair group method with arithmetic mean (UPGMA) developed by Sokal and Michener [46] to obtain dendrograms.

The Bayesian model-based clustering method was used for the determination of the genetic structure of the analyzed population using the STRUCTURE v. 2.2.4 software [47]. Samples were ordered according to the 16in1 and ECPGR dendrograms produced by POLYGENE, respectively. Ten independent runs were conducted for each K (1-26) with a burn-in length of 200,000 and 500,000 MCMC reps after burn-in. A Web version of StructureSelector (http://lmme.qdio.ac.cn/StructureSelector/, accessed on 10 November 2024, [48]), which implements the method developed by Evanno et al. [49], was used to evaluate K values for the analyzed data. Varieties with inferred clusters ≥ 0.95 and ≥0.90, respectively, for the group with the highest and the second highest delta K values, were assumed as representative varieties for individual groups.

The tanglegram was constructed in R for the 294 genotypes unique in the 16in1 system. Genetic distance matrices were calculated based on codominant SSR data using custom functions in R (version 2.5-7) [50]. Genotypic data were converted into genind objects using the adegenet package (version 2.1.10) [51]. Hierarchical clustering was performed using the hclust() function with average linkage, and dendrograms were visualized and compared using the dendextend package (version 1.17.1) [52]. Topological similarity was assessed via cophenetic correlation and the Mantel test implemented in the vegan package [53].

Parentage analysis for 294 genotypes unique in the 16in1 system was run in POLYGENE software, version V1.7 [44] by the Phenotype method, looking for parent pairs (sex unknown); selfing was permitted. The top 5 LOD score pairs with a null score of Trio loci mismatching were extracted.

## 5. Conclusions

Though thousands of cherry varieties exist, only a limited number of ancestor varieties have been used for breeding. This poses a challenge for their reliable identification, as many groups of cherry varieties are genetically very similar. Therefore, a new SSR marker set was developed, validated, and formulated in a one-tube PCR assay. Our 16in1 system showed a discrimination power seven orders of magnitude better than the currently used SSR marker sets, making it especially useful for parentage and population structure analyses.

## Figures and Tables

**Table 1:** Characteristics of newly identified SSR markers. PIC_HipSTR_: Polymorphic information content calculated by HipSTR tool on allele sequences.

SSR Marker	Chromosome	Position on ‘Tieton’, v2.0 Reference Genome	Location in Genomic Region	Repetition Motif	PIC_HipSTR_
Pav_chr1_028	1	56,439,028	intron of FUN_007320_hypothetical protein	AAT	0.721
Pav_chr1_073	1	52,114,073	3’UTR of FUN_006722_Kinesin-like protein KIN-7E	AT	0.829
Pav_chr2_274	2	44,310,274	intergenic region	CT	0.833
Pav_chr2_995	2	44,515,995	intergenic region	AG	0.855
Pav_chr3_002	3	24,943,002	intergenic region	CT	0.902
Pav_chr3_706	3	21,366,706	intergenic region	AG	0.835
Pav_chr3_765	3	5,316,765	intergenic region	TAT	0.711
Pav_chr4_217	4	3,209,217	intergenic region	GT	0.81
Pav_chr4_499	4	275,499	3’UTR of FUN_031522_hypothetical protein	TCTAGT	0.734
Pav_chr5_059	5	16,156,059	intron of FUN_023912_hypothetical protein	AT	0.853
Pav_chr5_144	5	32,811,144	intergenic region	CT	0.856
Pav_chr5_198	5	16,788,198	intergenic region	TTGC	0.640
Pav_chr5_903	5	32,741,903	intergenic region	AG	0.846
Pav_chr6_178	6	10,235,178	intron of FUN_019469_hypothetical protein	AG	0.825
Pav_chr6_363	6	37,421,363	intergenic region	AGA	0.749
Pav_chr6_387	6	25,964,387	intergenic region	AG	0.830
Pav_chr6_418	6	29,689,418	intergenic region	AGTT	0.691
Pav_chr6_505	6	3,361,505	intron of FUN_018452_hypothetical protein	AG	0.829
Pav_chr6_641	6	25,614,641	intergenic region	ATT	0.812
Pav_chr6_989	6	35,139,989	intergenic region	AG	0.854
Pav_chr7_259	7	23,664,259	intergenic region	CT	0.832
Pav_chr7_286	7	13,399,286	intron of FUN_037207_hypothetical protein	AT	0.825
Pav_chr7_444	7	19,590,444	intron of FUN_037952_hypothetical protein	CT	0.821
Pav_chr7_737	7	29,755,737	intergenic region	AAAG	0.786
Pav_chr7_798	7	12,815,798	intergenic region	AG	0.85
Pav_chr7_863	7	28,148,863	intergenic region	ATAA	0.648
Pav_chr7_867	7	23,682,867	intron of FUN_038587_hypothetical protein	CT	0.846
Pav_chr7_906	7	6,627,906	intron of FUN_036438_hypothetical protein	ATGT	0.666
Pav_chr8_224	8	29,212,224	intergenic region	AAT	0.762
Pav_chr8_438	8	25,182,438	intergenic region	CT	0.836

**Table 2:** Parameters of the newly identified SSR markers based on the analysis of 49 selected samples. k: Number of alleles; Ho: Observed heterozygosity; He: Expected heterozygosity; PIC: Polymorphic information content; Ae: Number of effective alleles; nt: Nucleotides. Notes: Reasons for not including the SSR marker in the final genotyping set.

SSR Marker	k	Ho	He	PIC	Ae	Minimal Length (nt)	Maximal Length (nt)	Notes
Pav_chr1_028	4	0.796	0.736	0.686	3.784	209	221	No amplification in multiplex
Pav_chr1_073 *	8	0.776	0.807	0.780	5.175	115	133	
Pav_chr2_274 *	7	0.878	0.837	0.816	6.125	415	438	
Pav_chr2_995	8	0.898	0.790	0.762	4.754	124	167	In close proximity to Pav_chr2_274
Pav_chr3_002 *	11	0.898	0.850	0.833	6.651	300	324	
Pav_chr3_706 *	10	0.918	0.827	0.804	5.772	221	247	
Pav_chr3_765	7	0.735	0.736	0.696	3.790	235	264	
Pav_chr4_217 *	5	0.898	0.747	0.709	3.952	406	433	
Pav_chr4_499 *	4	0.755	0.712	0.657	3.477	227	245	
Pav_chr5_059	11	0.816	0.846	0.828	6.507	215	243	No amplification in multiplex
Pav_chr5_144 *	9	0.796	0.826	0.804	5.744	332	362	
Pav_chr5_198	3	0.755	0.712	0.657	3.477	395	425	Low PIC, Ho, He, Ae
Pav_chr5_903	8	0.837	0.830	0.808	5.892	290	312	In close proximity to Pav_chr5_144
Pav_chr6_178 *	7	0.878	0.820	0.797	5.545	104	126	
Pav_chr6_363	7	0.816	0.760	0.724	4.168	121	141	
Pav_chr6_387	7	0.837	0.763	0.728	4.227	127	155	
Pav_chr6_418	5	0.816	0.711	0.655	3.460	177	208	
Pav_chr6_505 *	7	0.857	0.798	0.769	4.951	304	323	
Pav_chr6_641	9	0.915	0.798	0.769	4.953	410	465	No amplification in multiplex
Pav_chr6_989	6	0.857	0.720	0.682	3.570	250	281	Lower Ho, He, Ae
Pav_chr7_259	8	0.878	0.828	0.806	5.828	112	128	Lower He, PIC, Ae
Pav_chr7_286	9	0.851	0.826	0.805	5.753	132	152	No amplification in multiplex
Pav_chr7_444	6	0.714	0.713	0.671	3.480	417	455	
Pav_chr7_737	8	0.837	0.771	0.741	4.361	89	117	Lower Ho, He, Ae
Pav_chr7_798 *	8	0.837	0.831	0.809	5.928	308	330	
Pav_chr7_863	3	0.571	0.608	0.540	2.553	187	195	
Pav_chr7_867 *	9	0.878	0.838	0.818	6.180	423	460	
Pav_chr7_906	3	0.735	0.661	0.587	2.953	279	292	
Pav_chr8_224	10	0.796	0.790	0.763	4.759	197	255	No amplification in multiplex
Pav_chr8_438 *	8	0.776	0.831	0.808	5.907	386	416	

* SSR marker chosen for the final genotyping set.

**Table 3:** Primers used in the 16in1 system for the genotyping of sweet cherry; the type of fluorescent labeling is incorporated into the name of the corresponding primer.

Primer Name	Sequence (5’-3’)	Final Concentration in PCR (μM)
6-FAM-BPPCT037-F	CATGGAAGAGGATCAAGTGC	0.28
BPPCT037-R	CTTGAAGGTAGTGCCAAAGC	0.28
6-FAM-EMPA005-F	TGGGTTTGAGCAATATGCAACTG	0.27
EMPA005-R	CACCAATACACATGCACACGTAT	0.27
6-FAM-Pav_chr4_217-F	AAGGTGGTGGTGGTATCCTG	0.09
Pav_chr4_217-R	CCACTTGTCACTCACTCCAC	0.09
Pav_chr6_505-F	AAGAGGTGGAGAGGCATTCC	0.18
6-FAM-Pav_chr6_505-R	AACCATAGGAAGCCAAGCGC	0.18
Pav_chr1_073-F	GTAAAACTCCTGTGACCCAAATGT	0.65
VIC-Pav_chr1_073-R	GGCGGTATACAGAGAAGGCT	0.65
VIC-Pav_chr3_706-F	CTTGCTTGCTTTTCCTGTGTGA	0.12
Pav_chr3_706-R	GCCTCGCAATCAGATAGCAG	0.12
VIC-Pav_chr5_144-F	AGCCACTTGAAACCACATACGT	0.16
Pav_chr5_144-R	CACACAGGCACACAATCACAG	0.16
VIC-Pav_chr2_274-F	ATTAAGTAACTTTTGGGTTGGGTAAC	0.44
Pav_chr2_274-R	GTTATAACTTACATACATAACCGACC	0.44
NED-UCD-CH11-F	TGMTATTAGCTTAATGCCTCCC	0.4
UCD-CH11-R	ATGCTGATGTCATAAGGTGTGC	0.4
NED-Pav_chr4_499-F	TAACGGAATTGGAGCAAAGGGAA	0.09
Pav_chr4_499-R	CAAACAATGACCCACCTCCTG	0.09
NED-Pav_chr3_002-F	CCCAACTATTTATCCCATTGGCA	0.12
Pav_chr3_002-R	GACGAACGAAGGTACCATGC	0.12
NED-Pav_chr8_438-F	TGGCTCCAAAACAGAATGTGGAA	0.09
Pav_chr8_438-R	CTAGCTGCTGTCGTATCCCT	0.09
PET-Pav_chr6_178-F	AGGAAAGCTCACAATCAAGGGT	0.13
Pav_chr6_178-R	TATTCCACAAACACACACAACCC	0.13
PET-CPPCT006-F	AATTAACTCCAACAGCTCCA	0.5
CPPCT006-R	ATGGTTGCTTAATTCAATGG	0.5
PET-Pav_chr7_798-F	GGGGCGTTGTTCTATAGGCT	0.12
Pav_chr7_798-R	CAACTCTCACGTCGAAATGCC	0.12
PET-Pav_chr7_867-F	CCAACTAGGCTTCGGATTGC	0.095
Pav_chr7_867-R	ACCCGAAAGTTCCCATGACTC	0.095

**Table 4:** Results of statistical analysis for each marker using the 16in1 genotyping system (294 unique genotypes). k: Number of alleles; Ho: Observed heterozygosity; He: Expected heterozygosity; PIC: Polymorphic information content; Ae: Number of effective alleles.

SSR Marker	k	Ho	He	PIC	Ae
BPPCT037	11	0.867	0.804	0.775	5.101
EMPA005	10	0.633	0.647	0.606	2.832
Pav_chr4_217	5	0.759	0.702	0.662	3.352
Pav_chr6_505	9	0.786	0.806	0.779	5.160
Pav_chr1_073	11	0.827	0.825	0.802	5.705
Pav_chr3_706	13	0.884	0.824	0.802	5.677
Pav_chr5_144	10	0.891	0.845	0.826	6.457
Pav_chr2_274	11	0.837	0.823	0.799	5.642
UCD_CH11	11	0.810	0.799	0.769	4.980
Pav_chr4_499	4	0.776	0.735	0.685	3.770
Pav_chr3_002	13	0.867	0.862	0.846	7.223
Pav_chr8_438	9	0.857	0.825	0.802	5.724
Pav_chr6_178	10	0.816	0.829	0.806	5.850
CPPCT006	9	0.748	0.718	0.676	3.550
Pav_chr7_798	12	0.847	0.842	0.822	6.323
Pav_chr7_867	10	0.898	0.841	0.821	6.291
Average	9.875	0.819	0.795	0.767	5.227

**Table 5:** Results of statistical analysis for each marker using the ECPGR genotyping system (294 unique genotypes). k: Number of alleles; Ho: Observed heterozygosity; He: Expected heterozygosity; PIC: Polymorphic information content; Ae: Number of effective alleles.

SSR Marker	k	Ho	He	PIC	Ae
EMPaS12	7	0.765	0.754	0.713	4.068
EMPA003	4	0.466	0.436	0.342	1.773
EMPaS02	8	0.701	0.679	0.650	3.118
EMPA017	7	0.245	0.248	0.235	1.329
EMPaS10	14	0.588	0.598	0.562	2.488
EMPaS14	5	0.663	0.577	0.491	2.367
UDP98-412	8	0.643	0.623	0.595	2.656
EMPaS06	10	0.860	0.828	0.806	5.813
UDP98-410	8	0.527	0.525	0.476	2.104
EMPaS01	8	0.674	0.654	0.592	2.891
UDP98-411	7	0.762	0.708	0.666	3.419
EMPA026	3	0.609	0.581	0.491	2.387
EMPA002	4	0.609	0.493	0.373	1.971
CPPCT006	10	0.748	0.718	0.676	3.550
BPPCT037	12	0.867	0.804	0.775	5.101
CPPCT022	11	0.704	0.650	0.587	2.856
Average	7.875	0.652	0.617	0.564	2.994

**Table 6:** Comparison of statistical parameters in the ‘Van’ group based on results of the 16in1 system. Ho: Observed heterozygosity; He: Expected heterozygosity; PIC: Polymorphic information content; Ae: Number of effective alleles.

	Ho			He			PIC			Ae		
SSR Marker	All	K9.7 50%	K9.7 75%	All	K9.7 50%	K9.7 75%	All	K9.7 50%	K9.7 75%	All	K9.7 50%	K9.7 75%
BPPCT037	0.867	0.800	0.786	0.804	0.618	0.599	0.775	0.570	0.529	5.101	2.615	2.497
EMPA005	0.633	0.514	0.571	0.647	0.474	0.482	0.606	0.406	0.395	2.832	1.901	1.931
Pav_chr4_217	0.759	0.686	0.571	0.702	0.614	0.518	0.662	0.540	0.416	3.352	2.593	2.074
Pav_chr6_505	0.786	0.857	0.786	0.806	0.753	0.635	0.779	0.715	0.572	5.160	4.056	2.741
Pav_chr1_073	0.827	0.771	0.929	0.825	0.656	0.589	0.802	0.599	0.501	5.705	2.906	2.435
Pav_chr3_706	0.884	0.943	0.857	0.824	0.739	0.681	0.802	0.706	0.633	5.677	3.828	3.136
Pav_chr5_144	0.891	0.771	0.714	0.845	0.635	0.589	0.826	0.591	0.514	6.457	2.737	2.435
Pav_chr2_274	0.837	0.771	0.643	0.823	0.710	0.651	0.799	0.665	0.577	5.642	3.446	2.861
UCD_CH11	0.810	0.686	0.571	0.799	0.685	0.533	0.769	0.631	0.424	4.980	3.174	2.142
Pav_chr4_499	0.776	0.657	0.500	0.735	0.676	0.638	0.685	0.622	0.591	3.770	3.090	2.761
Pav_chr3_002	0.867	0.914	0.857	0.862	0.776	0.696	0.846	0.745	0.645	7.223	4.455	3.294
Pav_chr8_438	0.857	0.686	0.643	0.825	0.599	0.477	0.802	0.519	0.363	5.724	2.492	1.912
Pav_chr6_178	0.816	0.914	0.857	0.829	0.719	0.679	0.806	0.674	0.627	5.850	3.561	3.111
CPPCT006	0.748	0.171	0.071	0.718	0.161	0.069	0.676	0.156	0.067	3.550	1.192	1.074
Pav_chr7_798	0.847	0.886	0.714	0.842	0.796	0.735	0.822	0.767	0.688	6.323	4.910	3.769
Pav_chr7_867	0.898	0.857	0.857	0.841	0.690	0.635	0.821	0.652	0.561	6.291	3.228	2.741
Average	0.819	0.743	0.683	0.795	0.644	0.575	0.767	0.597	0.506	5.227	3.136	2.557

**Table 7:** Comparison of statistical parameters in the ‘Van’ family group based on results of the ECPGR system. Ho: Observed heterozygosity; He: Expected heterozygosity; PIC: Polymorphic information content; Ae: Number of effective alleles.

	Ho			He			PIC			Ae		
SSR Marker	All	K9.7 50%	K9.7 75%	All	K9.7 50%	K9.7 75%	All	K9.7 50%	K9.7 75%	All	K9.7 50%	K9.7 75%
EMPaS12	0.765	0.556	0.389	0.754	0.465	0.332	0.713	0.431	0.307	4.068	1.869	1.497
EMPA003	0.466	0.639	0.778	0.436	0.500	0.494	0.342	0.375	0.372	1.773	1.998	1.976
EMPaS02	0.701	0.381	0.167	0.679	0.388	0.204	0.650	0.370	0.194	3.118	1.633	1.256
EMPA017	0.245	0.028	0.001	0.248	0.027	0.001	0.235	0.027	0.001	1.329	1.028	1.001
EMPaS10	0.588	0.194	0.056	0.598	0.181	0.054	0.562	0.174	0.053	2.488	1.221	1.057
EMPaS14	0.663	0.667	0.722	0.577	0.585	0.619	0.491	0.510	0.541	2.367	2.411	2.623
UDP98-412	0.643	0.444	0.389	0.623	0.436	0.313	0.595	0.391	0.264	2.656	1.772	1.456
EMPaS06	0.860	0.830	0.795	0.828	0.791	0.768	0.806	0.760	0.731	5.813	4.785	4.319
UDP98-410	0.527	0.631	0.626	0.525	0.598	0.631	0.476	0.514	0.575	2.104	2.489	2.711
EMPaS01	0.674	0.544	0.506	0.654	0.581	0.526	0.592	0.494	0.439	2.891	2.388	2.107
UDP98-411	0.762	0.667	0.778	0.708	0.620	0.623	0.666	0.555	0.557	3.419	2.629	2.656
EMPA026	0.609	0.500	0.556	0.581	0.375	0.401	0.491	0.305	0.321	2.387	1.600	1.670
EMPA002	0.609	0.667	0.611	0.493	0.475	0.486	0.373	0.362	0.368	1.971	1.906	1.946
CPPCT006	0.748	0.250	0.111	0.718	0.229	0.106	0.676	0.220	0.104	3.550	1.297	1.119
BPPCT037	0.867	0.778	0.833	0.804	0.641	0.620	0.775	0.586	0.559	5.101	2.784	2.634
CPPCT022	0.704	0.583	0.556	0.650	0.471	0.444	0.587	0.383	0.346	2.856	1.891	1.800
Average	0.652	0.522	0.492	0.617	0.459	0.412	0.564	0.402	0.356	2.994	2.097	1.973

## Data Availability

The original contributions presented in this study are included in the article/Supplementary Material. Further inquiries can be directed to the corresponding author.

## Supplementary Materials

The following supporting information can be downloaded at: https://www.mdpi.com/article/10.3390/ijms27052324/s1.

## References

[1] Schuster M.M., Schröpfer S., Flachowsky H. (2024). An overview of the self-incompatibility (S) genotypes of cultivated sweet cherries. Acta Hortic..

[2] Herrero M., Rodrigo J., Wünsch A., Quero-García J., Iezzoni A., Puławska J., Lang G. (2017). Flowering, fruit set and development. Cherries: Botany, Production and Uses.

[3] Fadón E., Rodrigo J., Luedeling E. (2021). Cultivar-specific responses of sweet cherry flowering to rising temperatures during dormancy. Agric. For. Meteorol..

[4] İmrak B., Kafkas N.E., Çömlekçioğlu S., Bilgin Ö.F., Gölcü A.E., Burgut A., Attar Ş.H., Küçükyumuk C., Küçükyumuk Z. (2025). A Comparative Study of Dormex^®^ and Biostimulant Effects on Dormancy Release, Productivity, and Quality in ‘Royal Tioga^®^’ Sweet Cherry Trees (*Prunus avium* L.). Horticulturae.

[5] Wenden B., Campoy J.A., Jensen M., López-Ortega G., Quero-García J., Iezzoni A., Puławska J., Lang G. (2017). Climatic Limiting Factors: Temperature. Cherries: Botany, Production and Uses.

[6] Testolin R., Messina R., Cipriani G., De Mori G. (2023). SSR-based DNA fingerprinting of fruit crops. Crop Sci..

[7] Chambers A., Carle S., Njuguna W., Chamala S., Bassil N., Whitaker V., Barbazuk W., Folta K. (2013). A genome-enabled, high-throughput, and multiplexed fingerprinting platform for strawberry (*Fragaria* L.). Mol. Breed..

[8] Bassil N., Bidani A., Nyberg A., Hummer K., Rowland L. (2020). Microsatellite markers confirm identity of blueberry (*Vaccinium* spp.) plants in the USDA-ARS National Clonal Germplasm Repository collection. Genet. Resour. Crop Evol..

[9] Čmejlová J., Pluhařová K., Krška B., Čmejla R. (2025). A New Set of SSR Markers Combined in One Reaction for Efficient Genotyping of the Hexaploid European Plum (*Prunus domestica* L.). Plants.

[10] Nybom H., Lācis G. (2021). Recent Large-Scale Genotyping and Phenotyping of Plant Genetic Resources of Vegetatively Propagated Crops. Plants.

[11] Clarke J., Tobutt K. (2003). Development and characterization of polymorphic microsatellites from *Prunus avium* ‘Napoleon’. Mol. Ecol. Notes.

[12] Struss D., Ahmad R., Southwick S.M., Boritzki M. (2003). Analysis of Sweet Cherry (*Prunus avium* L.) Cultivars Using SSR and AFLP Markers. J. Am. Soc. Hortic. Sci..

[13] Vaughan S., Russell K. (2004). Characterization of novel microsatellites and development of multiplex PCR for large-scale population studies in wild cherry, *Prunus avium*. Mol. Ecol. Notes.

[14] Cipriani G., Lot G., Huang W.G., Marrazzo M.T., Peterlunger E., Testolin R. (1999). AC/GT and AG/CT microsatellite repeats in peach [*Prunus persica* (L) Batsch]: Isolation, characterisation and cross-species amplification in *Prunus*. Theor. Appl. Genet..

[15] Aranzana M.J., Garcia-Mas J., Carbo J., Arús P. (2002). Development and variability analysis of microsatellite markers in peach. Plant Breed..

[16] Dirlewanger E., Cosson P., Tavaud M., Aranzana M., Poizat C., Zanetto A., Arús P., Laigret F. (2002). Development of microsatellite markers in peach [*Prunus persica* (L.) Batsch] and their use in genetic diversity analysis in peach and sweet cherry (*Prunus avium* L.). Theor. Appl. Genet..

[17] Mnejja M., Garcia-Mas J., Howad W., Badenes M.L., Arús P. (2004). Simple-sequence repeat (SSR) markers of Japanese plum (*Prunus salicina* Lindl) are highly polymorphic and transferable to peach and almond. Mol. Ecol. Notes.

[18] Mnejja M., Garcia-Mas J., Howad W., Arús P. (2005). Development and transportability across prunus species of 42 polymorphic almond microsatellites. Mol. Ecol. Notes.

[19] Clarke J.B., Tobutt K.R. (2009). A standard set of accessions, microsatellites and genotypes for harmonising the fingerprinting of cherry collections for the ECPGR. Acta Hortic..

[20] Reim S., Schiffler J., Braun-Lüllemann A., Schuster M., Flachowsky H., Höfer M. (2023). Genetic and Pomological Determination of the Trueness-to-Type of Sweet Cherry Cultivars in the German National Fruit Genebank. Plants.

[21] Barreneche T., Cárcamo de la Concepción M., Blouin-Delmas M., Ordidge M., Nybom H., Lacis G., Feldmane D., Sedlak J., Meland M., Kaldmäe H. (2021). SSR-Based Analysis of Genetic Diversity and Structure of Sweet Cherry (*Prunus avium* L.) from 19 Countries in Europe. Plants.

[22] Ordidge M., Litthauer S., Venison E., Blouin-Delmas M., Fernandez-Fernandez F., Höfer M., Kägi C., Kellerhals M., Marchese A., Mariette S. (2021). Towards a Joint International Database: Alignment of SSR Marker Data for European Collections of Cherry Germplasm. Plants.

[23] Akin M., Nyberg A., Postman J., Mehlenbacher S., Bassil N. (2017). A multiplexed microsatellite fingerprinting set for hazelnut cultivar identification. Eur. J. Hortic. Sci..

[24] Zurn J.D., Nyberg A., Montanari S., Postman J., Neale D., Bassil N. (2020). A new SSR fingerprinting set and its comparison to existing SSR- and SNP-based genotyping platforms to manage *Pyrus* germplasm resources. Tree Genet. Genomes.

[25] Čmejlová J., Rejlová M., Paprštein F., Čmejla R. (2021). A new one-tube reaction kit for the SSR genotyping of apple (*Malus* × *domestica* Borkh.). Plant Sci..

[26] Marchese A., Giovannini D., Leone A., Mafrica R., Palasciano M., Cantini C., Di Vaio C., De Salvador F.R., Giacalone G., Caruso T. (2017). *S*-genotype identification, genetic diversity and structure analysis of Italian sweet cherry germplasm. Tree Genet. Genomes.

[27] Liu C., Qi X., Song L., Li Y., Li M. (2018). Species identification, genetic diversity and population structure of sweet cherry commercial cultivars assessed by SSRs and the gametophytic self-incompatibility locus. Sci. Hortic..

[28] Liu Y., Chang K., Zhu Y., Tang D., Chen W., Tang Q., Tan L. (2025). Development and Application of Tetra/Penta-Nucleotide SSR Markers for Paternal Identification in the Tea Plant (*Camellia sinensis)*. Plants.

[29] Zhang S., Li Y., Li Y., Zhang Y., Hao Y., Chen Y., Hou Z., Qi J. (2024). Identification and genetic diversity analysis of specific walnut F1 progeny based on SSR molecular markers: Taking heart-shaped walnuts and Jinghong 1 as examples. Sci. Rep..

[30] Vuletin Selak G., Baruca Arbeiter A., Cuevas J., Perica S., Pujic P., Raboteg Božiković M., Bandelj D. (2021). Seed Paternity Analysis Using SSR Markers to Assess Successful Pollen Donors in Mixed Olive Orchards. Plants.

[31] Choi C., Kappel F. (2004). Inbreeding, Coancestry, and Founding Clones of Sweet Cherries from North America. J. Am. Soc. Hortic. Sci..

[32] Campoy J.A., Lerigoleur-Balsemin E., Christmann H., Beauvieux R., Girollet N., Quero-García J., Dirlewanger E., Barreneche T. (2016). Genetic diversity, linkage disequilibrium, population structure and construction of a core collection of *Prunus avium* L. landraces and bred cultivars. BMC Plant Biol..

[33] Mariette S., Tavaud M., Arunyawat U., Capdeville G., Millan M., Salin F. (2010). Population structure and genetic bottleneck in sweet cherry estimated with SSRs and the gametophytic self-incompatibility locus. BMC Genet..

[34] Wiersma P.A., Erogul D., Ali S. (2018). DNA Fingerprinting of Closely Related Cultivars of Sweet Cherry. J. Am. Soc. Hortic. Sci..

[35] Fernandez i Marti A., Athanson B., Koepke T., Font i Forcada C., Dhingra A., Oraguzie N. (2012). Genetic diversity and relatedness of sweet cherry (*Prunus avium* L.) cultivars based on single nucleotide polymorphic markers. Front. Plant Sci..

[36] Palasciano M., Zuluaga D.L., Cerbino D., Blanco E., Aufiero G., D’Agostino N., Sonnante G. (2022). Sweet Cherry Diversity and Relationships in Modern and Local Varieties Based on SNP Markers. Plants.

[37] Quero Garcia J., Branchereau C., Barreneche T., Dirlewanger E. (2022). DNA-informed breeding in sweet cherry: Current advances and perspectives. Italus Hortus.

[38] Holušová K., Čmejlová J., Suran P., Čmejla R., Sedlák J., Zelený L., Bartoš J. (2023). High-resolution genome-wide association study of a large Czech collection of sweet cherry (*Prunus avium* L.) on fruit maturity and quality traits. Hortic. Res..

[39] Temnykh S., DeClerck G., Lukashova A., Lipovich L., Cartinhour S., McCouch S. (2001). Computational and experimental analysis of microsatellites in rice (*Oryza sativa* L.): Frequency, length variation, transposon associations, and genetic marker potential. Genome Res..

[40] Thiel T., Michalek W., Varshney R.K., Graner A. (2003). Exploiting EST databases for the development and characterization of gene-derived SSR-markers in barley (*Hordeum vulgare* L.). Theor. Appl. Genet..

[41] Wang J., Liu W., Zhu D., Hong P., Zhang S., Xiao S., Tan Y., Chen X., Xu L., Zong X. (2020). Chromosome-scale genome assembly of sweet cherry (*Prunus avium* L.) cv. Tieton obtained using long-read and Hi-C sequencing. Hortic. Res..

[42] Willems T., Zielinski D., Yuan J., Gordon A., Gymrek M., Erlich Y. (2017). Genome-wide profiling of heritable and de novo STR variations. Nat. Methods.

[43] Untergasser A., Cutcutache I., Koressaar T., Ye J., Faircloth B.C., Remm M., Rozen S.G. (2012). Primer3—New capabilities and interfaces. Nucleic Acids Res..

[44] Huang K., Dunn D.W., Ritland K., Li B.G. (2020). POLYGENE: Population genetics analyses for autopolyploids based on allelic phenotypes. Methods Ecol. Evol..

[45] Ayres K.L., Overall A.D.J. (2004). API-CALC 1.0: A computer program for calculating the average probability of identity allowing for substructure, inbreeding and the presence of close relatives. Mol. Ecol. Notes.

[46] Sokal R.R., Michener C.D. (1958). A statistical method for evaluating systematic relationships. Univ. Kans. Sci. Bull..

[47] Pritchard J.K., Stephens M., Donnelly P. (2000). Inference of population structure using multilocus genotype data. Genetics.

[48] Li Y.L., Liu J.X. (2018). StructureSelector: A web based software to select and visualize the optimal number of clusters using multiple methods. Mol. Ecol. Resour..

[49] Evanno G., Regnaut S., Goudet J. (2005). Detecting the number of clusters of individuals using the software STRUCTURE: A simulation study. Mol. Ecol..

[50] R Core Team (2021). R: A Language and Environment for Statistical Computing.

[51] Jombart T. (2008). adegenet: A R package for the multivariate analysis of genetic markers. Bioinformatics.

[52] Galili T. (2015). dendextend: An R package for visualizing, adjusting and comparing trees of hierarchical clustering. Bioinformatics.

[53] Oksanen J., Blanchet F.G., Friendly M., Kindt R., Legendre P., McGlinn D., Minchin P., O’Hara R.B., Simpson G., Solymos P. (2020). R Package.

